# Supplementary material for: Crawling and Gliding: A Computational Model for Shape-Driven Cell Migration
Source: PLoS Comput Biol. 2015 Oct 21;11(10):e1004280. doi: 10.1371/journal.pcbi.1004280 (PMC4619082; doi:10.1371/journal.pcbi.1004280)
Supplement: S1 Code — (ZIP) [file pcbi.1004280.s012.zip › release/tst/doc/html/warning_8h.html]

Tissue Simulation Toolkit: warning.h File Reference


Tissue Simulation Toolkit
 0.1.4.1


- Main Page
- Namespaces
- Classes
- Files

- File List
- File Members

Macros |
Functions

warning.h File Reference

This graph shows which files directly or indirectly include this file:

Go to the source code of this file.

|  |  |
| --- | --- |
| Macros | |
| #define | MEMORYCHECK(x)   if ((x)==NULL) { fprintf(stderr, "Out of Memory error in "#x" \n"); exit(0); } |
|  | |
| #define | UNIDENTIFIED   2353996 |
|  | |

|  |  |
| --- | --- |
| Functions | |
| void | error (char \*,...) |
|  | |
| void | warning (char \*,...) |
|  | |

## Macro Definition Documentation

|  |  |  |  |  |  |
| --- | --- | --- | --- | --- | --- |
| #define MEMORYCHECK | ( |  | x | ) | if ((x)==NULL) { fprintf(stderr, "Out of Memory error in "#x" \n"); exit(0); } |

Referenced by ReadLine().

#define UNIDENTIFIED   2353996

## Function Documentation

|  |  |  |  |
| --- | --- | --- | --- |
| void error | ( | char \* | , |
|  |  |  | *...* |
|  | ) |  |  |

Referenced by bgetpar(), CheckFile(), dgetparlist(), ParsePar(), ReadLine(), SearchToken(), and SkipToken().

|  |  |  |  |
| --- | --- | --- | --- |
| void warning | ( | char \* | , |
|  |  |  | *...* |
|  | ) |  |  |

References Quiet.

Referenced by bgetpar(), dgetparlist(), fgetpar(), igetpar(), ParsePar(), SearchToken(), and sgetpar().


---

Generated on Thu Aug 14 2014 22:04:01 for Tissue Simulation Toolkit by  

 1.8.6
